# Biomarkers of Inflammation and Inflammation-Related Indexes upon Emergency Department Admission Are Predictive for the Risk of Intensive Care Unit Hospitalization and Mortality in Acute Poisoning: A 6-Year Prospective Observational Study

**DOI:** 10.1155/2021/4696156

**Published:** 2021-08-19

**Authors:** Catalina Lionte, Cristina Bologa, Victorita Sorodoc, Ovidiu Rusalim Petris, Gabriela Puha, Alexandra Stoica, Alexandr Ceasovschih, Elisabeta Jaba, Laurentiu Sorodoc

**Affiliations:** ^1^Internal Medicine and Clinical Toxicology Department, Faculty of Medicine, University of Medicine and Pharmacy “Grigore T. Popa”, 700115 Iasi, Romania; ^2^2nd Internal Medicine Clinic, “Sf. Spiridon” Emergency Clinic County Hospital, 700111 Iasi, Romania; ^3^Internal Medicine and Nursing Department, Faculty of Medicine, University of Medicine and Pharmacy “Grigore T. Popa”, 700115 Iasi, Romania; ^4^Statistics Department, FEEA, “Alexandru Ioan Cuza” University, 700506 Iasi, Romania

## Abstract

Patients poisoned with drugs and nonpharmaceutical substances are frequently admitted from the emergency department (ED) to a medical or ICU department. We hypothesized that biomarkers of inflammation and inflammation-related indexes based on the complete blood cell (CBC) count can identify acutely poisoned patients at increased risk for ICU hospitalization and death. We performed a 6-year prospective cohort study on 1548 adult patients. The demographic data, the levels of hs-CRP (high-sensitivity C-reactive protein), CBC, and inflammation-related indexes based on CBC counts were collected upon admission and compared between survivors and nonsurvivors, based on the poison involved. Both a multivariate logistic regression model with only significant univariate predictors and a model including univariate predictors plus each log-transformed inflammation-related indexes for mortality were constructed. The importance of the variables for mortality was graphically represented using the nomogram. hs-CRP (odds ratio (OR), 1.38; 95% CI, 1.16–1.65, *p* < 0.001 for log-transformed hs-CRP), red cell distribution width (RDW), neutrophil-lymphocyte ratio (NLR), and platelet-lymphocyte ratio (PLR) were significantly associated with the risk of ICU hospitalization, after multivariable adjustment. Only RDW, NLR, and monocyte-lymphocyte ratio (MLR) were significantly associated with mortality. The predictive accuracy for mortality of the models which included either NLR (AUC 0.917, 95% CI 0.886-0.948) or MLR (AUC 0.916, 95% CI 0.884-0.948) showed a high ability for prognostic detection. The use of hs-CRP, RDW, NLR, and MLR upon ED admission are promising screening tools for predicting the outcomes of patients acutely intoxicated with undifferentiated poisons.

## 1. Introduction

Acute poisonings represent an important cause of mortality and a challenge to the Emergency Department (ED) in many countries. The majority of cases presented to the ED are self-poisonings, and the substances which have been causing severe outcomes for the past decade are antidepressants, stimulants and street drugs, antihistamines, and anticonvulsants [1]. Nevertheless, acute toxicities after pesticide exposure globally account for the overwhelming majority of poisoning deaths [2]. Up to 40% of the patients visiting the ED with an intoxication are admitted to the hospital, and an average of 1.5–3.7% poisoned patients need intensive care unit (ICU) admission [3]. There were studies which, based on hematologic parameters, such as the complete blood count (CBC), red blood cell distribution width (RDW), and neutrophil-lymphocyte ratio (NLR), attempted to predict the outcomes of patients poisoned with carbon monoxide (CO), paraquat, organophosphates (OPs), lead, or mushrooms [4–9].

However, appropriate risk stratification using biomarkers of inflammation and inflammation-related indexes based on peripheral CBC counts measured in the ED for the need of ICU hospitalization and in-hospital mortality remains a challenge in poisoning with both pharmaceutical and nonpharmaceutical agents. On many occasions, patients are brought in the ED with an altered mental status after being exposed to a xenobiotic, and it is difficult to make a quick prognosis assessment, especially when a toxicological screen or a quantitative measurement of the poison involved is not available or is delayed. High sensitivity CRP, CBC, and inflammation-related indexes based on CBC are readily available in the ED and inexpensive, but no studies have evaluated the prognostic value of these parameters in patients admitted to the hospital with acute poisonings with undifferentiated toxins.

The primary aim of this study was to investigate whether biomarkers of inflammation and inflammation-related indexes based on peripheral CBC counts measured in the ED are associated with the outcomes in patients hospitalized for acute poisoning with pharmaceutical agents, nonpharmaceutical substances, and combination of poisons.

## 2. Materials and Methods

### 2.1. Study Design and Setting

This prospective, observational cohort study was conducted in a university hospital, a tertiary referral center for acute poisonings in North-Eastern Romania. The study was conducted between January 1, 2015, and December 31, 2020. Approval from the local University and Hospital Ethics Committee was obtained for the study, which was conducted in compliance with the guidelines of the Helsinki Declaration. The study complied with the transparent reporting of an observational cohort study (STROBE) and with a multivariable prediction model for individual prognosis (TRIPOD) statement [10].

### 2.2. Selection of Participants

Acutely poisoned adult patients were included in the study and were defined as patients admitted by the ED with accidental or self-poisoning with undifferentiated xenobiotics, including pharmaceutical agents (prescription drugs and over-the-counter (OTC) drugs), street drugs, nonpharmaceutical substances (toxic alcohols and chemicals, OPs, organochlorine pesticides, carbamates, rodenticides, and caustic substances), toxic gases (CO, cyanide, and arsenic), plant toxins (poisonous mushrooms, Aconitum, Datura stramonium, Atropa belladonna, and Nerium oleander), and a combination of poisons within 24 hours of exposure. The exclusion criteria consisted of age (patients younger than 17), pregnancy, known hematologic disease, previous chemotherapy treatment (within the last month), blood transfusion (within the last 2 weeks), a history of autoimmune disease, liver cirrhosis, trauma, burns, temperature more than 37.5°C or ongoing acute infection, discharge against the doctor's orders, and transfer before the final outcome was determined. The following data were collected: age, sex, comorbidities, the body mass index (BMI), the laboratory results upon presentation, the time interval from the poison exposure to the ED arrival, the intentionality of the poisoning, the Glasgow Coma Scale (GCS) score and vital signs upon presentation, CBC counts (neutrophils, monocytes, lymphocytes, and platelets) upon presentation and other biochemistry tests, repeated afterwards upon physician request, and the duration of hospital stay in a medical or ICU department.

### 2.3. Outcome Measures

The primary aim of this study was to investigate whether biomarkers of inflammation and inflammation-related indexes based on peripheral CBC counts are associated with the need for ICU care, development of complications, and in-hospital mortality and which scores might significantly improve the predictive accuracy for the outcomes in patients acutely poisoned with undifferentiated poisons, upon ED admission. We analyzed respiratory, cardiovascular, hepatorenal, gastroenteral, hematological, metabolic, and CNS complications developed as a direct consequence of intoxication. In order to find out exactly how soon after the poisoning these scores have a prognostic value, we described the changes in the patient's biomarkers of inflammation and inflammation-related indexes in relation with the poison type, within 24 hours of acute exposure.

### 2.4. Blood Analysis

Complete blood counts and differentials were studied in the peripheral blood samples: white blood cell (WBC) count, neutrophils, lymphocytes, monocytes, hemoglobin (Hb), platelets, and red cell distribution width (RDW) upon ED admission. Blood samples were taken in calcium-EDTA tubes. CBC were performed with Sysmex XT-4000i-Automated Hematology Analyzer (Sysmex Corporation, Tokyo, Japan). Inflammation-related indexes based on peripheral CBC counts were calculated as follows: the systemic immune inflammation index (SII) = platelet count × neutrophil count/lymphocyte count; the neutrophil‐lymphocyte ratio (NLR) = neutrophil count/lymphocyte count; the monocyte‐lymphocyte ratio (MLR) = monocyte count/lymphocyte count; and the platelet‐lymphocyte ratio (PLR) = platelet count/lymphocyte count. Arterial blood gases, hs-CRP, and other biochemistry parameters were obtained using ABL 90 (Radiometer, Denmark) and ARCHITECT c16000 clinical chemistry analyzer (Abbott Laboratories, Abbott Park, Illinois, USA).

### 2.5. Data Analysis

Statistical analysis was performed using SPSS version 22.0 for Windows (IBM SPSS, Chicago, IL, United States) and STATA 13.0 statistical software (StataCorp, College Station, Texas, United States). Descriptive variables are expressed as the mean ± SD for data that are normally distributed and as the median and interquartile range (IQR) for variables that are not normally distributed. The *χ*^2^ or Fisher exact test was used to compare categorical values, expressed as percentages. For continuous variables, Student's *t* test or the Mann–Whitney test was used for two group comparisons according to normality. Variables found to be significant in univariate analysis, regarding their correlation with mortality, with a *p* value of <0.05 were subjected to multivariate logistic regression analysis.

The following variables, which can be easily evaluated upon ED presentation, were tested in the univariate analysis: age, hs-CRP, initial GCS score, arterial lactate, and RDW. In our model, we used RDW-SD (expressed in fL), which is an actual measurement of the width of the red blood cell (RBC) size distribution histogram, because it is not influenced by the average RBC size, as is the situation with RDW-CV [11]. The first multivariate logistic model included significant univariate predictors (model 1). The significant univariate predictors and NLR were entered into a second multivariate logistic regression model (model 2). Then, NLR in model 2 was successively replaced in subsequent models with the SII (model 3), PLR (model 4), or MLR (model 5). For the multivariate logistic analysis, the NLR, SII, PLR, and MLR were logarithmically transformed using the base logarithm of 2 because of their positively skewed and wide distribution. To avoid multicollinearity, each multivariate model (models 2-5) included one score and other significant univariate predictors. Before modelling, if two or more variables in univariate analysis retained in the multivariate analysis were highly correlated in the linear regression, one variable was removed to avoid collinearity. Estimated odds ratios (ORs) and 95% CIs were calculated for all significant variables. The diagnostic performance of each regression model and each parameter was assessed using receiver operating characteristic (ROC) curves and the corresponding areas under the curve performance. The importance of the effects of clinical and laboratory variables for mortality was graphically represented using the nomogram [12]. The nomogram is a visualization of a complex model equation, with the aim of representing the behavior of a predictor in scales [13]. Kattan-style nomograms were generated in Stata using the nomolog program for binary logistic models [14].

## 3. Results

### 3.1. Baseline Characteristics

**Figure 1:**
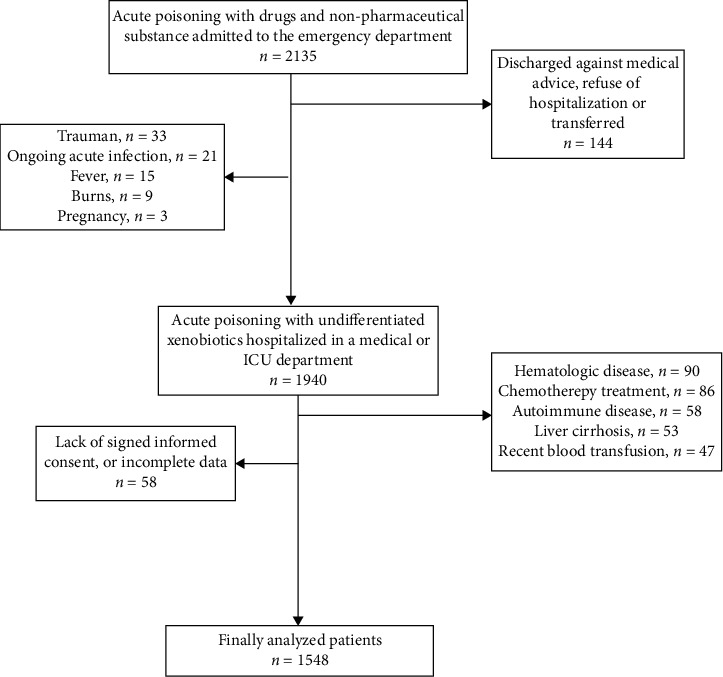
Study flow diagram.

We included 1548 patients with a median age of 46 years (range 17-98) who presented to our hospital's ED at a mean of 5 hours (range 30 min to 24 hours) after exposure to a drug, a nonpharmaceutical substance, or a combination of poisons (Figure 1).

The baseline characteristics of the cohort are presented in Table 1. 316 patients (20.41%) were hospitalized in the ICU, 1072 patients (69.3%) developed complications, and fifty-nine patients (3.8%) died during hospitalization. Associated comorbidities consisted of psychiatric conditions (30.3%), cardiovascular diseases (25.3%), addictions (12.5%), renal diseases (3.6%), respiratory diseases (3.4%), gastrointestinal and hepatobiliary illnesses (4.8%), and diabetes (1.6%). 25.2% of all patients had an abnormal BMI, but this was not correlated with mortality. Based on BMI, no significant differences in leukocytes, platelet count, Hb levels, NLR, MLR, SII, and PLR were recorded.

### 3.2. hs-CRP and CBC Count in Relation with the Outcomes

When comparing the hs-CRP and CBC count, the nonsurvivor group had higher hs-CRP, RDW, WBC, lymphocyte, and monocyte counts upon presentation in the ED (Table 1).

High sensitivity CRP, RDW, WBC, neutrophil, and monocyte counts were significantly associated (*p* ≤ 0.001) with ICU hospitalization (Supplementary Table S1).

Compared to the patients who had no complications, the patients who developed complications during hospitalization had significantly higher hs-CRP, RDW, WBC, neutrophil, and monocyte counts upon presentation in the ED (Supplementary Table S2).

The analysis of these parameters based on the group of poisons showed that hs-CRP was significantly higher in patients with caustics poisoning compared with patients intoxicated with combination of poisons, OTC drugs, street drugs, toxic alcohols and chemicals, pesticides, and plant toxins (Supplementary Table S3).

The WBC counts were also significantly higher in pesticides, caustics and toxic alcohols, and chemicals poisoning compared with values recorded in poisoning with a combination of toxins, prescription drugs, and OTC drugs (Supplementary Table S4).

**Figure 2:**
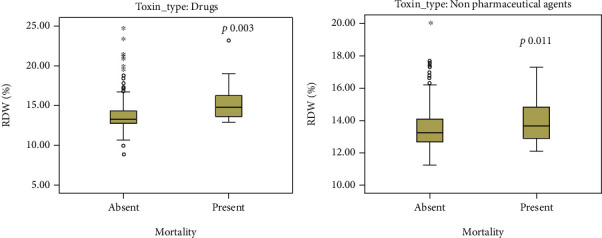
Box plot demonstrating the effect of admission RDW on mortality in patients poisoned with pharmaceutical agents (a) and in patients poisoned with nonpharmaceutical substances (b). Values are median and interquartile range; dots represent outliers; ∗ represent extreme values.

Regarding RDW, values recorded were significantly higher in poisoning with prescription drugs compared with combination of toxins, pesticides, OTC medications, caustic substances, and plant toxin poisoning (Supplementary Table S4). RDW was significantly higher in nonsurvivors poisoned with pharmaceutical agents and nonpharmaceutical substances (Table 2, Figure 2). Interestingly, RDW values were higher in poisoning with toxic gases (13.82 ± 1.48), as opposed to combination of toxins (13.32 ± 1.59, *p* = 0.009), pesticides (13.35 ± 0.99, *p* = 0.031), OTC medications (13.27 ± 1.36, 0.037), plant toxins (13.18 ± 0.83, 0.019), and caustic poisoning (13.20 ± 1.35, *p* = 0.006).

### 3.3. Inflammation-Related Indexes Based on CBC Count and Outcomes

The nonsurvivor group had significantly higher NLR, MLR, and SII values within 24 hours of poison exposure than the survivor group (Table 3).

**Figure 3:**
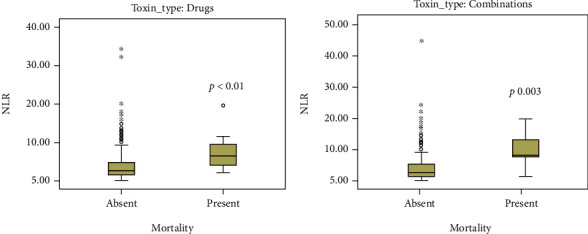
Box plot demonstrating the effect of admission NLR on mortality in patients poisoned with pharmaceutical agents (a) and in patients poisoned with combination of poisons (b). Values are median and interquartile range; dots represent outliers; ∗ represent extreme values.

The analysis based on the main type of the poison involved revealed that NLR, SII, and MLR had significantly higher values in nonsurvivors poisoned with pharmaceutical agents and combinations, while PLR was significantly higher in nonsurvivors poisoned with pharmaceutical agents and nonpharmaceutical substances (Table 2, Figure 3, Figure S1, figure S2).

We attempted to correlate each type of drug with the specific alterations in serum markers in nonsurvivors vs. survivors. Among nonsurvivors acutely intoxicated with pharmaceutical agents, cardiovascular drugs were responsible for 15.3%, followed by sedative-hypnotics (5.1%), antiepileptics (3.4%), and antidepressants (1.7%). In our cohort, we identified significant differences between hs-CRP, RDW, NLR, and MLR in cardiovascular drugs poisoning resulting in death, between hs-CRP, NLR, and MLR in poisoning with combinations of drugs/toxins and also between NLR and MLR in deceased patients poisoned with sedative-hypnotics compared with survivors. hs-CRP was significantly higher in nonsurvivors poisoned with antiepileptic drugs (Supplementary Table S5). No significant correlations were found in antidepressant drugs poisoning, where almost all patients survived (only one deceased patient).

NLR had higher values recorded in poisoning with caustics, plant toxins, pesticides, compared with poisoning with prescription drugs, or a combination of toxins (Supplementary Table S4). Also, significant differences were recorded between NLR in toxic gases poisoning (6.68 ± 7.59), compared with poisoning involving prescription drugs (3.63 ± 3.16, *p* = 0.016). SII was significantly higher in poisoning with caustics, plant toxins, pesticides and toxic gases compared with poisoning with prescription drugs (Supplementary Table S4). We noticed significant differences in SII values recorded in poisoning with caustic substances (2216.29 ± 3907.97) as compared with poisoning with toxic alcohols and chemicals (1394.42 ± 1830.62, *p* = 0.011), OTC drugs (1213.89 ± 1240.62, *p* = 0.014), and combination of toxins (1036.18 ± 1237.80, *p* < 0.001). PLR was significantly increased in poisoning with plant toxins, toxic gases, caustics, and pesticides compared with prescription drug overdoses (Supplementary Table S4)). MLR had significantly higher values in poisoning with caustics, plant toxins, pesticides, and toxic gases, compared with prescription drug overdoses (Supplementary Table S4). Also, MLR recorded in poisoning with caustic substances (0.55 ± 0.99) was higher compared with poisoning with OTC drugs (0.27 ± 0.31, *p* < 0.001), pesticides (0.39 ± 0.45, *p* = 0.04), toxic alcohols and chemicals (0.33 ± 0.44, *p* < 0.001), and combination of toxins (0.24 ± 0.25, *p* < 0.001).

Patients with in-hospital complications had significantly higher values of NLR, PLR, SII, and MLR compared with patients with no complications recorded (Table 3).

We also analyzed CBC parameters predictive for complications in a multivariate analysis, and only RDW and MLR showed a predictive value for this outcome (Table 4).

As for the need of ICU hospitalization, only GCS score < 8, hs-CRP, RDW, and NLR were predictive for this outcome in univariate and multivariate analysis (Table 5).

**Figure 4:**
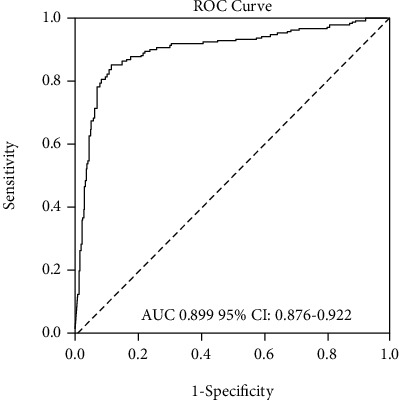
Receiver operating characteristic (ROC) curves compared the diagnostic accuracy of the model predicting the need for ICU hospitalization.

The model including hs-CRP, RDW, and NLR upon ED arrival had a significantly higher predictive accuracy for the need of ICU hospitalization (AUC 0.899 [0.876-0.922], *p* < 0.001, Figure 4).

Because we aimed to determine which scores have better prognostic value for mortality, the CBC-based scores upon ED presentation were examined by univariate and multivariate analyses (Table 6). hs-CRP did not correlate with mortality in multivariate analysis, so it was excluded from the final model. To avoid collinearity, the ratios of differential WBC counts (NLR, PLR, SII, and MLR) were entered into different models.

**Figure 5:**
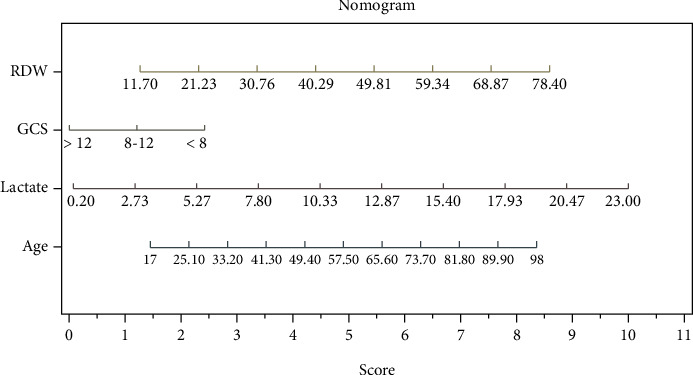
Nomogram constructed for model 1 included age, arterial lactate upon ED arrival, GCS score, and RDW.

**Figure 6:**
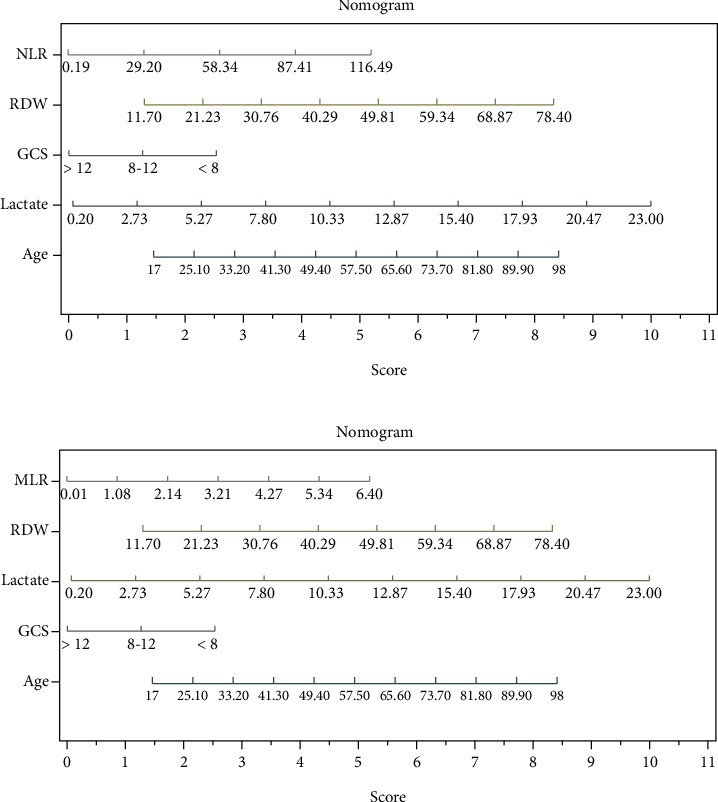
(a) Nomogram constructed for model 2 included all variables in model 1 and NLR. (b) Nomogram constructed for model 5 included all variables in model 1 and MLR.

Age, initial GCS score < 8, arterial lactate, and RDW, which were identified in the univariate analysis as independent predictors for mortality, were tested in the multivariate analysis. In models including each score and the univariate factors, among the scores based on the peripheral CBC count at presentation, only NLR and MLR were significantly associated with mortality (Table 6). The importance of each variable for mortality was graphically shown using the nomogram. Nomograms were built for each model, and the diagrams are presented in Figure 5 for model 1, Figure 6(a) for model 2 and Figure 6(b) for model 5. The nomogram which compared NLR, SII, and PLR is presented in supplementary Figure S3. In the nomogram construction, only the explanatory variables with an important influence on the mortality of the poisoned patients were kept.

**Figure 7:**
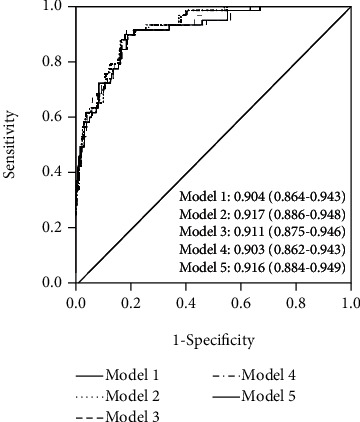
Receiver operating characteristic (ROC) curves compares the diagnostic accuracy of the five models constructed (AUC and 95% CI are presented for each model).

Comparing the AUCs for each model revealed that the model including the NLR upon ED presentation plus parameters in model 1 (model 2 AUC 0.917 [0.886-0.948]), and the model including the MLR upon ED arrival plus parameters in model 1 (model 5 AUC 0.916 [0.884-0.948]) had a significantly higher predictive accuracy for mortality than the model including RDW alone (model 1 AUC 0.904 [0.864-0.943]; *p* < 0.001, Figure 7).

## 4. Discussion

Better resource allocation for intoxicated patients in ED will prevent unnecessary admissions to the hospital or ICU, taking into account hospital understaffing and increased number of patients. Also, it will increase the availability of ICU care for those patients that really need ICU treatment, and it will reduce costs [3]. To create a better allocation, it is necessary to identify and manage the complicated patients from readily available parameters and accurate prognostic scoring systems, as well as improving benchmarking indices to predict the need for ICU hospitalization, development of complications, and in-hospital mortality when applied to the emergency setting. This study investigated the predictive performance of inflammation biomarkers and inflammation-related indexes measured in the ED in patients acutely poisoned with undifferentiated xenobiotics. The hs-CRP and complete blood count are routinely determined in all patients presenting with acute poisoning to the ED. These results are usually quick and inexpensive. Several studies revealed the value of hematological parameters in predicting short- or long-term mortality in patients with acute myocardial infarction [15], sepsis and septic shock [16], acute pancreatitis [17], or in critically ill patients [18]. Also, there were studies which analyzed either hs-CRP or hematological parameters in relation with short-term outcomes in poisoning: leukocyte, neutrophil counts, and NLR in paraquat poisoning [19], NLR in mushroom [20] and CO poisoning [21], hs-CRP [22], and RDW in organophosphate poisoning [5]. In CO poisoning, it was revealed that there is a correlation between RDW and long-term outcomes [3] and between SII and neurological outcomes [23]. This is the first study to investigate the relation between the inflammation biomarkers and inflammation-related indexes based on CBC count upon ED arrival with short-term outcomes in patients poisoned with undifferentiated drugs and nonpharmaceutical substances. The main findings of our study revealed that patients with a need for ICU hospitalization had higher hs-CRP, leukocyte, neutrophil, and monocyte counts, as well as higher dispersion of RBC within 24 hours of exposure to a xenobiotic. In addition, inflammation-related indexes calculated from the peripheral CBC count at presentation were independently associated with short-term outcomes after poisoning with xenobiotics, and among these, only the RDW, NLR, and MLR in combination with clinical and laboratory parameters significantly improved prognostic accuracy for in-hospital mortality and complications. The higher risk patients appeared to be elderly, with increased levels of hs-CRP, RDW, NLR, MLR, and lactate within 24 hours of exposure to a poison. Because these scores are easily measurable upon presentation in the ED, this study could facilitate a quick risk stratification in clinical practice for short-term outcomes among patients with drugs and nonpharmaceutical substances acute poisoning.

Plasma CRP level may be useful for the prediction of prognosis in paraquat poisoning [24], and the difference in C-reactive protein value between initial and follow-up after 24 hours was associated with mortality in a study which included 96 subjects with acute organophosphate poisoning [25]. However, our study showed that hs-CRP has a predictive role only for ICU hospitalization but not for mortality in a larger cohort of patients poisoned with undifferentiated drugs and nonpharmaceutical substances, although there are significant differences in values recorded upon ED presentation based on the group of toxins involved.

Red blood cell distribution width is a measure of the variability in the size of circulating erythrocytes. Although RDW has traditionally been used for the diagnosis of different types of anemia, recent studies reported that RDW is a strong predictor of morbidity and mortality in various clinical conditions, including cardiovascular diseases, community-dwelling older adults, or general in-hospital patients [26]. Acute exposure to various medication increases the risk of adverse drug reactions and toxicity and might lead to high size variation and increased RDW value. We identified higher values of RDW in acute intoxication with prescription drugs. An elevated RDW is associated with several inflammatory markers, and proinflammatory cytokines could suppress the growth of RBC and decrease the half-life of RBC, which consequently produces an increased RDW [26]. Another mechanism that can explain the higher levels of RDW in patients who have exposure to different xenobiotics can be related to the acute effect of abnormal hemoglobin molecules on erythrocytes. Carboxyhemoglobin may cause anisocytosis and RDW elevation by making structural changes in erythrocytes [27]. Besides the acute effect of CO poisoning, hypoxia is the most important stimulant for increasing erythrocyte production [3]. Sulfhemoglobin, which persists for as long as the cell lives, is formed by irreversible oxidation of hemoglobin by drugs (i.e., sulfanilamides, phenacetin, and nitrites) or exposure to sulfur chemicals in industrial or environmental settings [28]. Also, many drugs and chemicals can induce methemoglobin formation (i.e., chloroquine, nitroprusside, sulfonamides, organic and inorganic nitrites and nitrates, aromatic amines, and chlorobenzene), as well as some fertilizers and herbicides [29]. We also found significantly higher RDW values in acute exposures to toxic gases. Although OPs can induce the formation of free radicals that interact with blood cells by changing hematological parameters [30], the patients with pesticide exposure in our cohort did not have a significantly higher value of RDW, compared with the other groups of poisons analyzed. Oxidative stress is the major mechanism in the pathophysiology of most toxins and diseases [31]. Experimental studies reported that erythrocyte fragility is increased due to the lipid peroxidation of the erythrocyte membrane in cases of severe poisoning, with increased oxidative stress burden, thus increasing the fragility of RBCs and shortening the life-span of RBCs [26, 32]. We tried to avoid other conditions influencing RDW values by excluding the patients whose associated diseases had a well-known relation with RDW, such as liver dysfunction, nutritional deficiencies, bone marrow dysfunction, inflammatory diseases, and chronic or acute systemic inflammation [5]. We consider that in our study, hemoglobin levels of patients poisoned with undifferentiated xenobiotics measured within 24 hours of exposure were not correlated with the outcomes possibly due to nondepleted antioxidant capacity of erythrocytes in the early period of poisoning. Another explanation could be the exclusion of the patients with comorbidities which might affect the Hb and RDW levels from the analysis. However, the RDW cannot provide physicians with accurate information on the inflammatory state and indication of the prognosis of patients with no other inflammatory indicators [33], so we thought that it was important to analyze other inflammation-related indexes based on CBC count for this purpose.

The NLR is a combination of 2 independent markers of inflammation: neutrophils, as a marker of ongoing nonspecific inflammation, and lymphocytes, as a marker of the regulatory pathway [21]. In pesticide poisoning, the sensitivity of erythrocytes and lymphocytes to oxidative stress depends on the balance between oxidative stress and antioxidant defense capacity [34]. NLR has been proven to be a useful prognostic factor in many diseases, such as neoplastic disease, stroke, and cardiovascular disease [35, 36]. NLR has also been established as a good indicator of systemic inflammatory status in the general population [37]. Neutrophils are well-known potential biomarkers of inflammation. Since inflammation is responsible for the pathogenic mechanism of tissues injury after poisoning with pesticides, caustic substances, toxic gases or toxic alcohols, and chemicals, the significantly higher neutrophil and NLR values after these acute exposures are not surprising. NLR is more stable than the neutrophil count alone because the neutrophil count is easily affected by infection, stress, or medication, which makes the change in the neutrophil count less informative [35, 38]. It has been extensively indicated that toxicity induced by herbicides is due to a sustained redox-cycling and the subsequent generation of reactive oxygen species, resulting in a general inflammatory reaction [19]. An increase in leukocytes and neutrophil counts and a decline in lymphocyte counts are observed when the CBC is evaluated during the acute inflammatory response caused by oxidative stress [39]. The inflammatory and hypoxemic effects of several xenobiotics included in our study might cause a stimulus in the bone marrow and probably induce a release of immature cells or an increase of other cells in the bloodstream, similar with other diseases [40]. Leukocytosis, neutrophilia, and monocytosis can be detected on CBC in the acute period of the clinical course when the oxidative stress is increased [41]. In our population, NLR and MLR were significantly higher in severely poisoned patients, who did not survive, as well as in patients who developed complications over the course of hospitalization. SII and PLR are commonly used inflammation-related indexes, and we recorded higher values of these parameters in patients poisoned with caustics, pesticides, toxic gases, and vegetal toxins compared with drug overdoses. However, the prognostic accuracy of the models including the SII and PLR in our cohort was not better compared with the models including NLR and MLR. This is in opposition with the results of other studies on SII and PLR, which showed a prognostic accuracy in cancer patients [42, 43], as well as a good predictive role for neurological long-term complications in CO-poisoned patients [23], probably because we were interested only in short-term outcomes and we did not analyze the long-term effect of these indices. Thrombocytopenia might appear after the oxidative stress which negatively affects the platelet membranes, as it does in all blood cells. Thrombocytopenia is frequently observed in nonpoisoning clinical conditions such as sepsis and pneumonia, where the oxidative stress is increased [41]. Our results also showed a significant lower platelet count in nonsurvivors after exposure to different xenobiotics. This might explain the poor value of SII and PLR as predictive variables for mortality and complications in this population. Survivors presented higher platelet counts than nonsurvivors in our population, which is in line with other reports on patients hospitalized in the ICU [44]. Although some poisons, such as CO, induce thrombocytosis and increased platelet activation and the platelets elicit a role in inflammation [23], this was not enough to produce a substantial effect when a wide range of poisons, with different mechanisms of action, is analyzed.

Key strengths of our study are the prospective design, the large sample size with a large number of poisons and outcome events, the high proportion of patients with complete data, and the availability of the biomarkers of inflammation and inflammation-related indexes analyzed. The main limitation of our study was that it was single-centered. However, our institution is located in northeastern Romania, it is a teaching university hospital and a tertiary referral center for acute poisoning, and therefore it could be used as a representative institution in this region. Another limitation was that the CBC-based parameters were analyzed only at the time of the patients' presentation and were not repeated afterwards in all patients. Additionally, confounding factors interfering with CBC counts might not have been completely excluded, as it is known that diet, exercise, smoking, vitamin supplements, or hormones influence RDW values [45]. Biomarkers of inflammation and inflammation-related indexes only reflect some aspects of the mechanism of acute poisoning with xenobiotics. We might assume that these parameters together with other potential prognostic biomarkers may be more reliable for the evaluation of the short-term outcomes of acute poisoning with pharmaceutical agents and nonpharmaceutical substances upon ED presentation. These findings must be confirmed in prospective, multicenter studies with larger populations.

## 5. Conclusions

The present study shows that acute poisoning with undifferentiated drugs, nonpharmaceutical substances, and combination of toxins can cause high levels of biomarkers of inflammation and inflammation-related indexes. hs-CRP, RDW, and NLR have a good prognostic value to predict the need for ICU hospitalization. Only RDW and inflammation-related indexes based on the CBC count, such as NLR and MLR were strongly associated with in-hospital mortality in acutely poisoned patients. Biomarkers of inflammation and inflammation-related indexes derived from the CBC, obtained in an automated way, simple and inexpensive, need to be valued as a daily tool for evaluating prognosis in hospitalized patients with poisoning involving undifferentiated drugs and nonpharmaceutical substances.

## Figures and Tables

**Table 1:** Baseline characteristics of acutely poisoned patients according to mortality.

Variables	Total (*n* = 1548)	Survivors (*n* = 1489)	Nonsurvivors (*n* = 59)	*p* value
Age (years)	46 [34-62]	46 [33-61]	66 [54-76]	<0.001
Gender (male, %)	729 (47.1)	706 (47.4)	23 (39)	0.127
Intentional exposure (%)	1095 (70.8)	1054 (70.8)	37 (62.7)	0.182
Poison involved (%)				<0.001
(i) Prescription drugs	483 (31.2)	468 (31.4)	15 (25.4)	
(ii) Combinations	401 (25.9)	391 (26.3)	10 (13.9)	
(iii) Over-the-counter drugs	67 (4.3)	67 (4.5)	0 (0)	
(iv) Street drugs	35 (2.3)	35 (2.4)	0 (0)	
(v) Toxic alcohols & chemicals	148 (9.6)	129 (8.7)	19 (32.2)	
(vi) Pesticides	144 (9.3)	138 (9.3)	6 (10.2)	
(vii) Caustic substances (acids, alkali)	125 (8.1)	120 (8.1)	5 (8.5)	
(viii) Toxic gases	86 (5.6)	82 (5.5)	4 (6.8)	
(ix) Plant toxins	59 (3.8)	59 (4.0)	0 (0)	
GCS score < 8 (%)	354 (22.9)	320 (21.5)	34 (57.6)	<0.001
SaO2 (%)	95.89 ± 5.87	96.12 ± 5.11	90.37 ± 14.43	<0.001
HR (b/min)	85 [74-100]	85 [74-100]	90 [75-118]	0.045
SBP (mmHg)	128 [110-142]	128 [111-142]	111 [80-134]	<0.001
Lactate (mmol/L)	1.9 [1.2-3.0]	1.89 [1.2-2.9]	6.6 [1.7-10.4]	<0.001
K+ (mmol/L)	4.0 [3.7-4.38]	4.0 [3.7-4.3]	4.4 [3.7-5.4]	0.001
hs-CRP (mg/dL)	0.37 [0.11-1.49]	0.35 [0.11-1.37]	2.24 [0.26-7.15]	<0.001
WBC (∗1000/mcgL)	9.21 [7.03-12.09]	9.13 [6.91-11.94]	13.21 [9.21-17.47]	<0.001
Lymphocytes (∗1000/mcgL)	2.27 ± 1.41	2.23 ± 1.31	3.08 ± 2.96	<0.001
Monocytes (∗1000/mcgL)	0.36 [0.25-0.52]	0.36 [0.25-0.51]	0.53 [0.33-0.76]	<0.001
Platelets (∗100000/mcgL)	243 [200-286]	244.5 [202-287]	221 [173-268]	0.009
Hb (g/dL)	13.51 ± 1.93	13.52 ± 1.90	13.23 ± 2.65	0.253
RDW-CV (%)	13.2 [12.6-14.1]	13.2 [12.6-14.1]	13.7 [12.9-15.3]	<0.002
RDW-SD (fL)	42.5 [40.2-45.7]	42.4 [40.1-45.3]	48.0 [42.4-51.9]	<0.001
Creatinine (mg/dL)	0.78 [0.70-0.92]	0.77 [0.70-0.90]	1.22 [1.00-1.84]	<0.001
ALAT (U/L)	20 [14-33]	20 [14-33]	31 [16-50]	<0.001
Need for ICU therapy (%)	316 (20.5)	262 (17.7)	54 (91.5)	<0.001
Hospitalization (days)	4 [3-6]	4 [3-6]	7 [2-12]	0.004

Data are presented as median [25–75 percentile], or percentage; GCS: Glasgow Coma Scale; HR: heart rate; SBP: systolic blood pressure; hs-CRP: high sensitivity C-reactive protein; WBC: white blood cells; Hb: hemoglobin; RDW: red cell distribution width; ALAT: alanine aminotransferase; ICU: intensive care unit.

**Table 2:** Correlation between admission CBC parameters with the poison type involved.

Poison type	CBC parameter	Survivors (*n* = 391)	Nonsurvivors (*n* = 10)	*p* value
Combination of poisons	WBC	9.62 ± 4.48	13.27 ± 2.72	0.001
	NLR	4.05 ± 4.37	9.67 ± 6.37	0.003
	RDW	13.35 ± 1.55	13.30 ± 2.52	0.444
	SII	1006.15 ± 1201.15	2204.20 ± 2000.29	0.022
	PLR	136.65 ± 86.94	194.83 ± 181.83	0.577
	MLR	0.23 ± 0.24	0.54 ± 0.35	0.003
Pharmaceutical agents	WBC	9.23 ± 3.93	8.94 ± 3.15	0.895
	NLR	3.83 ± 3.71	7.44 ± 4.55	<0.001
	RDW	13.74 ± 1.80	15.36 ± 2.81	0.003
	SII	941.66 ± 943.15	1592.20 ± 975.89	0.001
	PLR	137.60 ± 83.50	243.24 ± 151.82	0.003
	MLR	0.22 ± 0.21	0.42 ± 0.26	<0.001
Nonpharmaceutical substances	WBC	11.56 ± 5.26	16.62 ± 7.80	<0.001
	NLR	7.53 ± 10.95	8.13 ± 15.44	0.194
	RDW	13.44 ± 1.14	14.17 ± 1.64	0.011
	SII	1859.70 ± 2717.59	1828.02 ± 3373.69	0.105
	PLR	187.85 ± 197.95	121.37 ± 186.02	<0.001
	MLR	0.42 ± 0.60	0.45 ± 0.84	0.109

Data are presented as the mean ± standard deviation. CBC: complete blood count; WBC: white blood cells; NLR: neutrophil-lymphocyte ratio; RDW: red cell distribution width; SII: systemic immune inflammation index; PLR: platelet-lymphocyte ratio; MLR: monocyte-lymphocyte ratio.

**Table 3:** Inflammation-related indexes based on CBC count analyzed in respect of mortality and complications.

	Survivors	Nonsurvivors	*p* value	No complication	Any complication	*p* value
NLR	2.96 [1.72-5.58]	5.01 [2.26-8.90]	0.007	2.57 [1.56-4.29]	3.20 [1.80-6.62]	<0.001
PLR	119.22 [82.85-178.53]	102.68 [47.74-171.13]	0.598	114.81 [83.16-158.99]	121.21 [81.08-185.12]	0.001
SII	699.70 [393.57-1398.20]	904.98 [416.47-2034.96]	0.030	635.41 [361.25-1035.38]	745.55 [414.41-1693.42]	<0.001
MLR	0.17 [0.10-0.33]	0.28 [0.12-0.49]	0.015	0.15 [0.09-0.26]	0.19 [0.11-0.37]	<0.001

NLR: neutrophil-lymphocyte ratio; SII: systemic immune inflammation index; PLR: platelet-lymphocyte ratio; MLR: monocyte-lymphocyte ratio.

**Table 4:** Selected factors predictive for complications using univariate and multivariate analysis.

Variable	Univariate logistic regression			Multivariate logistic regression		
	OR	95% CI	*p* value	OR	95% CI	*p* value
Age	1.007	1.001-1.013	0.019	1.006	0.999-1013	0.117
Lactate	1.204	1.122-1.292	<0.001	1.129	1.048-1.215	0.001
GCS score < 8	0.800	0.764-0.838	<0.001	0.104	0.063-0.172	<0.001
RDW	3.890	1.456-10.392	0.007	2.889	0.869-9.602	0.083
NLR	1.452	1.277-1.651	<0.001	0.104	0.010-1.028	0.053
SII	1.355	1.203-1.526	<0.001	1.479	0.910-2.405	0.114
PLR	1.144	0.966-1.355	0.119	0.482	0.326-0.712	<0.001
MLR	1.501	1.313-1.715	<0.001	5.201	1.618-16.719	0.006

OR: odds ratio; CI: confidence interval; GCS: Glasgow Coma Scale; RDW: red cell distribution width; NLR: neutrophil-lymphocyte ratio; SII: systemic immune inflammation index; PLR: platelet-lymphocyte ratio; MLR: monocyte-lymphocyte ratio.

**Table 5:** Findings of the univariate and multivariate analysis predictive for ICU hospitalization.

	Univariate analysis		Multivariate analysis	
	Odds ratio (95% CI)	*p*	Odds ratio (95% CI)	*p*
Age	1.005 (0.999-1.012)	0.116	1.002 (0.989-1.015)	0.769
hs-CRP	1.354 (1.197-1.533)	≤0.001	1.387 (1.162-1.657)	≤0.001
NLR	1.219 (1.087-1.367)	0.001	2.384 (1.709-3.326)	≤0.001
RDW	1.270 (1.127-1.431)	≤0.001	1.382 (1.133-1.685)	0.001
Coma	0.024 (0.017-0.034)	≤0.001	0.016 (0.010-0.024)	≤0.001
Comorbidities present	0.455 (0.309-0.670)	≤0.001	0.602 (0.316-1.147)	0.123
PLR	1.016 (0.900-1.148)	0.794	0.444 (0.300-0.659)	≤0.001

**Table 6:** Univariate and multivariate logistic regression to identify independent predictors for mortality used in the five models.

Age	Lactate	GCS score < 8	RDW	Ln NLR	Ln SII	Ln PLR	Ln MLR
Univariate (OR [95% CI])							
1.05 (1.03-1.06)	1.35 (1.27-1.43)	0.20 (0.126-0.34)	1.14 (1.09-1.18)	1.39 (1.06-1.83)	1.19 (0.91-1.55)	0.64 (0.43-0.96)	1.37 (1.03-1.81)
Multivariate (adjusted OR [95% CI])							
1.06 (1.04-1.09)	1.37 (1.27-1.47)	0.17 (0.09-0.34)	1.08 (1.03-1.14)	**—**	**—**	**—**	**—**
1.06 (1.04-1.09)	1.37 (1.27-1.48)	0.16 (0.08-0.30)	1.08 (1.03-1.14)	1.47 (1.08-1.99)	**—**	**—**	**—**
1.06 (1.04-1.08)	1.37 (1.27-1.47)	0.17 (0.09-0.32)	1.08 (1.03-1.15)	**—**	1.23 (0.93-1.63)	**—**	**—**
1.06 (1.04-1.09)	1.36 (1.26-1.47)	0.18 (0.09-0.35)	1.08 (1.03-1.14)	**—**	**—**	0.96 (0.64-1.46)	**—**
1.06 (1.04-1.08)	1.37 (1.27-1.48)	0.16 (0.08-0.31)	1.08 (1.02-1.15)	**—**	**—**	**—**	1.43 (1.04-1.96)

GCS: Glasgow Coma Scale; RDW: red cell distribution width; Ln NLR: logarithmically transformed neutrophil-lymphocyte ratio; Ln SII: logarithmically transformed systemic immune inflammation index; Ln PLR: logarithmically transformed platelet-lymphocyte ratio; Ln MLR: logarithmically transformed monocyte-lymphocyte ratio; OR: odds ratio; CI: confidence interval.

## Data Availability

Data used to support the findings of this study are included within the article and supplementary information file.

## Supplementary Materials

Supplementary Materials The following are available: Table S1: baseline inflammation biomarkers and related indexes analyzed between the ICU hospitalization group and the non-ICU hospitalization group. Table S2: variables significantly associated with complications' development in poisoned patients. Table S3: significant differences in hs-CRP levels recorded based on the poison type. Table S4: changes in RDW and CBC-derived scores based on the poison type. Figure S1: box plot demonstrating the effect of admission MLR on mortality in patients poisoned with combination of poisons and pharmaceutical agents. Figure S2: box plot demonstrating the effect of admission PLR on mortality in patients poisoned with pharmaceutical and nonpharmaceutical agents. Figure S3: nomogram constructed using age, arterial lactate upon ED arrival, GCS score, RDW, NLR, SII, and PLR shows clear the lower significance of SII and PLR compared with NLR. Table S5: specific alterations in inflammation markers based on the type of drug in nonsurvivors and survivors' groups.
