# Supplementary material for: Biomarkers of Inflammation and Inflammation-Related Indexes upon Emergency Department Admission Are Predictive for the Risk of Intensive Care Unit Hospitalization and Mortality in Acute Poisoning: A 6-Year Prospective Observational Study
Source: Dis Markers. 2021 Aug 19;2021:4696156. doi: 10.1155/2021/4696156 (PMC8390135; doi:10.1155/2021/4696156)
Supplement: Supplementary Materials — The following are available: Table S1: baseline inflammation biomarkers and related indexes analyzed between the ICU hospitalization group and the non-ICU hospitalization group. Table S2: variables significantly associated with complications' development in poisoned patients. Table S3: significant differences in hs-CRP levels recorded based on the poison type. Table S4: changes in RDW and CBC-derived scores based on the poison type. Figure S1: box plot demonstrating the effect of admission MLR on mortality in patients poisoned with combination of poisons and pharmaceutical agents. Figure S2: box plot demonstrating the effect of admission PLR on mortality in patients poisoned with pharmaceutical and nonpharmaceutical agents. Figure S3: nomogram constructed using age, arterial lactate upon ED arrival, GCS score, RDW, NLR, SII, and PLR shows clear the lower significance of SII and PLR compared with NLR. Table S5: specific alterations in inflammation markers based on the type of drug in nonsurvivors and survivors' groups. [file 4696156.f1.docx]

Table S1. Baseline inflammation biomarkers and related indexes analyzed between the ICU hospitalization group and the non-ICU hospitalization group

| **Variable** | **ICU hospitalization group (n=316)** | **Non-ICU hospitalization group (n=1232)** | **p-value** |
| --- | --- | --- | --- |
| Hs-CRP (mg/dL) | 0.67[0.10-3.14] | 0.32[0.11-1.15] | <0.001 |
| RDW-SD (fL) | 43.4[40.4-47.4] | 42.4[40.1-45.3] | 0.001 |
| WBC(*1000/mcgL) | 10.53[7.69-15.93] | 8.93[6.85-11.51] | <0.001 |
| Neutrophils (*1000/mcgL) | 7.19[4.59-12.20] | 6.03[4.24-8.71] | <0.001 |
| Monocytes (*1000/mcgL) | 0.43[0.28-0.70] | 0.34[0.25-0.49] | <0.001 |

Data are presented as median [25–75 percentile]

Table S2. Variables significantly associated with complications’ development in poisoned patients

| **Variable** | **Complications group (n=1072)** | **Non-complications group (n=476)** | **p-value** |
| --- | --- | --- | --- |
| Hs-CRP (mg/dL) | 2.28±0.17 | 1.19±0.18 | 0.006 |
| RDW-CV (%) | 13.57±1.59 | 13.43±1.64 | 0.025 |
| WBC(*1000/mcgL) | 10.89±5.27 | 8.84±3.25 | <0.001 |
| Neutrophils (*1000/mcgL) | 7.98±4.97 | 6.05±3.08 | <0.001 |
| Monocytes (*1000/mcgL) | 0.46±0.27 | 0.35±0.17 | <0.001 |

Data are presented as mean ± SD

Table S3. Significant differences in hs-CRP levels recorded based on the poison type

| **Poison** | **hs-CRP (mg/dL)** | **p-value** |
| --- | --- | --- |
| Caustic substances | 3.29±5.63 | - |
| Combination of poisons | 1.65±4.06 | 0.003 |
| OTC drugs | 0.69±3.7 | 0.001 |
| Street drugs | 0.49±0.56 | 0.007 |
| Toxic alcohols and chemicals | 1.66±3.59 | 0.01 |
| Pesticides | 1.75±4.88 | 0.019 |
| Plant toxins | 1.18±2.69 | 0.008 |

Data are presented as mean ± SD

| **Poison type** | **WBC** | **Poison type** | **WBC** | **p-value** |
| --- | --- | --- | --- | --- |
| Pesticides  Caustics  Toxic alcohols and chemicals  Pesticides | 12.70±6.63  11.97±5.02  11.81±5.92  12.70±6.63 | Combinations  Prescription drugs  OTC drugs  Plant toxins | 9.69±4.43  9.09±3.99  8.86±2.79  10.36±4.32 | <0.001  0.001  0.044 |
| **Poison type** | **NLR** | **Poison type** | **NLR** | **p-value** |
| Caustics  Plant toxins  Pesticides | 9.83±18.13  8.73±8.73  6.95±8.09 | Prescription drugs  Combinations | 3.63±3.16  4.20±4.46 | < 0.001 |
| **Poison type** | **SII** | **Poison type** | **SII** | **p-value** |
| Caustics  Plant toxins  Pesticides  Toxic gases | 2216.29±3907.97  2163.72±2627.99  2006.19±2751.37  1696.44±2048.77 | Prescription drugs | 881.56±829.80 | <0.001  0.007 |
| **Poison type** | **PLR** | **Poison type** | **PLR** | **p-value** |
| Plant toxins  Toxic gases  Caustics  Pesticides | 236.43±239.45  198.67±225.71  193.86±242.73  178.07±169.18 | Prescription drugs | 134.80±83.63 | <0.001  0.003  0.001  0.04 |
| **Poison type** | **MLR** | **Poison type** | **MLR** | **p-value** |
| Caustics  Plant toxins  Pesticides  Toxic gases | 0.55±0.99  0.50±0.49  0.39±0.45  0.38±0.42 | Prescription drugs | 0.21±0.18 | <0.001  <0.001  <0.001  0.017 |
| **Poison type** | **RDW-CV** | **Poison type** | **RDW-CV** | **p-value** |
| Prescription drugs | 13.86±1.95 | Combinations  Pesticides  OTC medications  Caustics  Plant toxins | 13.32±1.59  13.35±0.99  13.28±1.36  13.20±1.35  13.18±0.83 | <0.001  0.027  0.005  0.002  0.002 |

Table S4. Changes in RDW and CBC-derived scores based on the poison type.

| 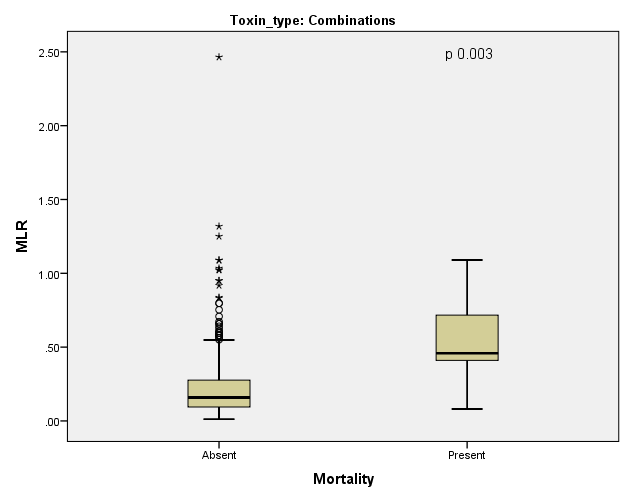 | 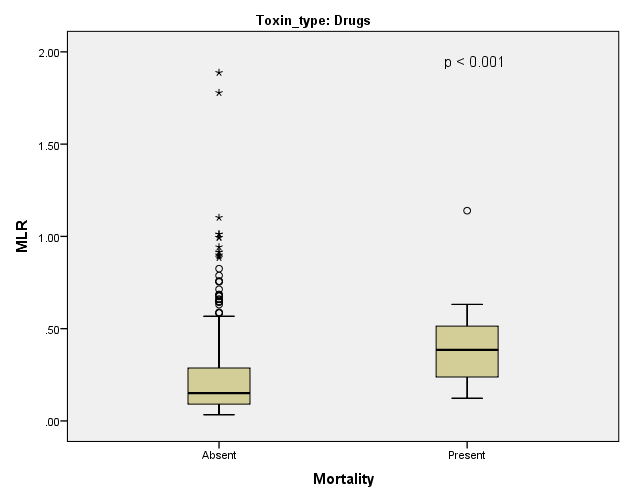 |
| --- | --- |
| (**a**) | (**b**) |

Figure S1: Box plot demonstrating the effect of admission MLR on mortality in patients poisoned with combination of poisons **(a)**, and in patients poisoned with pharmaceutical agents **(b)**. Values are median and interquartile range; dots represent outliers; * represent extreme values.

| 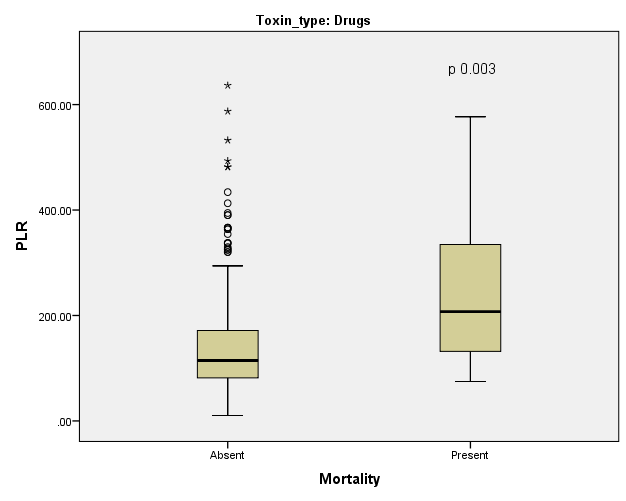 | 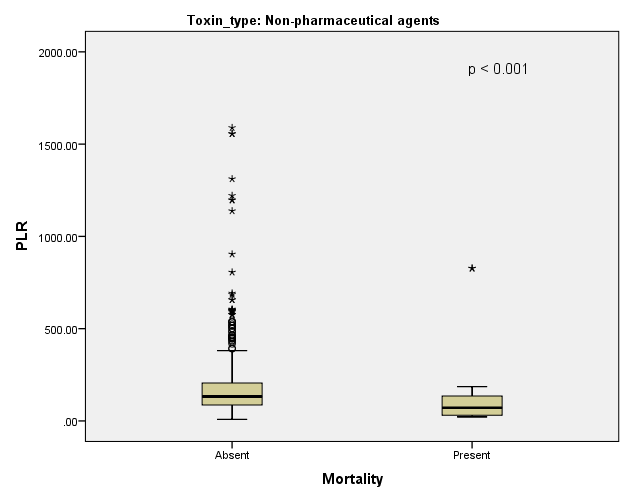 |
| --- | --- |
| (**a**) | (**b**) |

Figure S2: Box plot demonstrating the effect of admission PLR on mortality in patients poisoned with pharmaceutical agents **(a)**, and in patients poisoned with non-pharmaceutical substances **(b)**. Values are median and interquartile range; dots represent outliers; * represent extreme values.


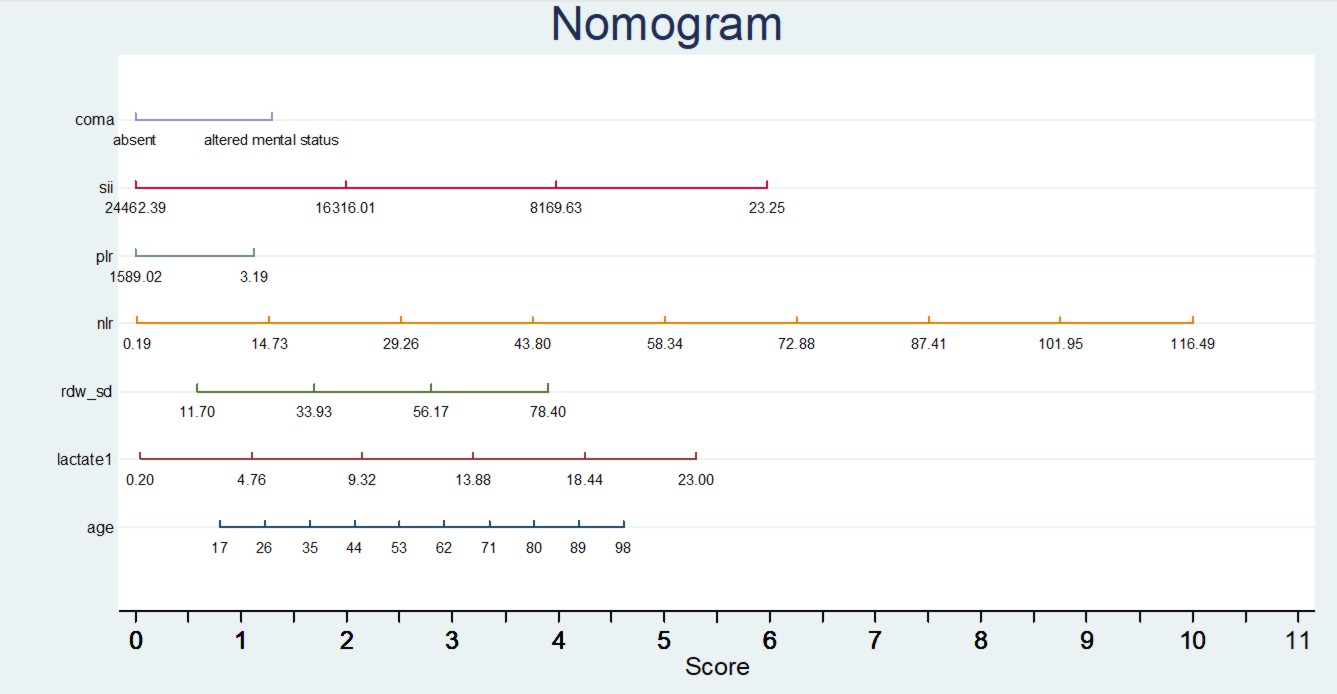


Figure S3: Nomogram constructed using age, arterial lactate upon ED arrival, coma (defined as GCS <8), RDW-SD, NLR, SII and PLR shows clear the lower significance of SII and PLR compared with NLR.

Table S5. Specific alterations in inflammation markers based on the type of drug in non-survivors and survivors’ groups.

| **Variable** | **Non-survivors** | **Survivors** | **p-value** |
| --- | --- | --- | --- |
| **Cardiovascular drugs poisoning** | | | |
| CRP (mg/dL) | 5.42±5.09 | 2.85±5.25 | 0.025 |
| RDW (fL) | 53.98±10.08 | 46.02±5.35 | 0.006 |
| NLR | 6.93±5.26 | 4.06±2.59 | 0.011 |
| MLR | 0.41±0.30 | 0.24±0.15 | 0.010 |
| **Combination of drugs/toxins poisoning** | | | |
| CRP (mg/dL) | 3.89±2.54 | 1.61±4.12 | < 0.001 |
| RDW (fL) | 45.11±6.80 | 42.65±4.70 | 0.725 |
| NLR | 10.28±6.36 | 4.23±4.45 | 0.003 |
| MLR | 0.57±0.35 | 0.24±0.24 | 0.003 |
| **Sedative-hypnotics poisoning** | | | |
| CRP (mg/dL) | 3.53±5.38 | 1.19±2.27 | 0.500 |
| RDW (fL) | 45.57±0.40 | 43.12±3.89 | 0.145 |
| NLR | 10.67±1.54 | 3.01±3.45 | 0.007 |
| MLR | 0.58±0.08 | 0.17±0.19 | 0.007 |
| **Antiepileptics poisoning** | | | |
| CRP (mg/dL) | 19.12±21.74 | 2.68±6.35 | 0.046 |
| RDW (fL) | 45.20±6.08 | 42.39±6.95 | 0.494 |
| NLR | 7.53±1.65 | 3.63±3.27 | 0.073 |
| MLR | 0.42±0.09 | 0.21±0.18 | 0.079 |

Data are presented as mean ± SD
